# In Situ Metal‐Oxygen‐Hydrogen Modified B‐Tio_2_@Co_2_P‐X S‐Scheme Heterojunction Effectively Enhanced Charge Separation for Photo‐assisted Uranium Reduction

**DOI:** 10.1002/advs.202305439

**Published:** 2023-12-04

**Authors:** Fucheng Zhang, Huanhuan Dong, Yi Li, Dengjiang Fu, Lu Yang, Yupeng Shang, Qiuyang Li, Yuwen Shao, Wu Gang, Tao Ding, Tao Chen, Wenkun Zhu

**Affiliations:** ^1^ State Key Laboratory of Environment‐friendly Energy Materials, National Co‐innovation Center for Nuclear Waste Disposal and Environmental Safety, Sichuan Co‐Innovation Center for New Energetic Materials, Nuclear Waste and Environmental Safety Key Laboratory of Defense, School of National Academy of Defense Technology, School of Life Science and Engineering Southwest University of Science and Technology 59 Qinglong Street Mianyang Sichuan 621010 P. R. China; ^2^ School of Materials and Energy University of Electronic Science and Technology Chengdu 610000 P.R. China; ^3^ University of Science and Technology of China National Synchrotron Radiation Laboratory Hefei 230029 P. R. China

**Keywords:** M‐O‐H, photocatalysis, S‐scheme heterojunction, titanium dioxide, uranium reduction

## Abstract

Photo‐assisted uranium reduction from uranium mine wastewater is expected to overcome the competition between impurity ions and U(VI) in the traditional process. Here, B‐TiO_2_@Co_2_P‐X S‐scheme heterojunction with metal‐oxygen‐hydrogen (M‐O‐H) is developed insitu modification for photo‐assisted U(VI) (hexavalent uranium) reduction. Relying on the DFT calculation and Hard‐Soft‐Acid‐Base (HSAB) theory, the introduction of metal‐oxygen‐hydrogen (M‐O‐H, hard base) metallic bonds in the B‐TiO_2_@Co_2_P‐X is found to enhance the hydrophilicity and the capture capability for uranyl ion (hard acid). Accordingly, B‐TiO_2_@Co_2_P‐500 hybrid nanosheets exhibit excellent U(VI) reduction ability (>98%) in the presence of competing ions. By self‐consistent energy band calculations and in‐situ KPFM spectral analysis, the formation of the internal electric field between B‐TiO_2_ and Co_2_P at the heterojunction is proven, offering a strong driving force and atomic transportation highway for accelerating the S‐scheme charge carriers directed migration and promoting the photocatalytic reduction of uranium. This work provides a valuable route to explore the functionally modified photocatalyst with high‐efficiency photoelectron separation for U(VI) reduction.

## Graphical abstract

A modified S‐scheme heterojunction B‐TiO_2_@Co_2_P‐X with in situ Metal‐Oxygen‐Hydrogen (M‐O‐H) is proposed to enhance hydrophilicity and capture capability of hexavalent uranium by overcoming competition from impurity ions in uranium mine wastewater. Additionally, the formation of an internal electric field at the heterojunction between B‐TiO_2_ and Co_2_P provides a strong driving force and atomic transportation pathway for photo‐assisted uranium reduction.

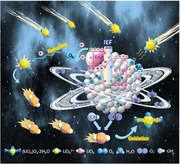

## 1 Introduction

During the process of uranium mining, a substantial quantity of radioactive wastewater containing uranium is generated, resulting in irreversible environmental damage and incurable harm to the surrounding ecological chain.^[^ ^1^, ^2^, ^3^, ^4^ ^]^ Uranium mine wastewater contains a large number of competitive metal cations, such as Ca^2+^, Mg^2+^, and so on, which will compete with U(VI) for active sites, making it difficult to recover uranium resources from uranium mine wastewater by most treatment methods. Photocatalysis is an efficient and green technology with natural selectivity to non‐valence ions, which can avoid ion interference and achieve reduction fixation of U (VI) in a multi‐ion coexistence environment.^[^ ^5^, ^6^, ^7^, ^8^, ^9^, ^10^, ^11^, ^12^, ^13^, ^14^, ^15^ ^]^ As a traditional photocatalyst, TiO_2_ is widely used in photocatalytic reduction of U(VI) due to its advantages of low cost, non‐toxic and harmless, and good chemical stability. However, the photocatalytic activity of traditional photocatalysts (TiO_2_) for reducing U (VI) is severely limited due to their weak response to visible light, lack of coordination of active sites, low concentration of photo‐generated carriers, etc.^[^ ^16^, ^17^ ^]^ At present, traditional photocatalysts (TiO_2_) modification methods mainly include doping,^[^ ^18^ ^]^ surface defect engineering,^[^ ^19^ ^]^ and heterojunction construction.^[^ ^20^ ^]^ Although these strategies can address certain issues present in traditional photocatalysts, simultaneously meeting the requirements of a narrow band gap, efficient separation holes, and abundant active sites poses significant challenges. Additionally, the reduction effect of U(VI) remains suboptimal. Therefore, exploring advanced photocatalysts with rich active sites and suitable energy band structures is a feasible route to achieve efficient photo‐assisted uranium reduction from uranium mine wastewater.^[^ ^21^, ^22^, ^23^ ^]^

Fortunately, the recently emerging S‐scheme heterojunction is considered a promising heterojunction system to ensure visible light absorption, enhance charge separation, and retain strong charge carrier redox capabilities.^[^ ^24^, ^25^, ^26^, ^27^, ^28^, ^29^ ^]^ For example, Wu et al.^[^ ^30^ ^]^ constructed an S‐scheme heterojunction of Bi_2_Sn_2_O_7_ quantum dots/TiO_2_ nanotube array to achieve efficient photoelectric degradation of sulfadiazine. Parallel to the construction of S‐scheme heterojunction, the enhancement of uranium binding ability usually originates from the tailored coordination sites with specific recognition for uranyl ion (hard acid) by designing the coordination environment of photocatalyst due to the Hard‐Soft‐Acid‐Base (HSAB) theory, which can be achieved simply by introducing metal‐oxygen‐hydrogen (M‐O‐H, hard base) metallic bonds. For instance, Liu et al.^[^ ^31^ ^]^ induced the formation of hydrogen‐metal bonds in WS_2_ by constructing defect engineering to enhance the selectivity of uranium. Therefore, the integration of S‐scheme heterojunction and M‐O‐H metallic bonds provides a potential strategy to improve the capture and reduction of uranium by engineering the electronic structure of photocatalysts and thereby enhancing the efficiency of photo‐assisted uranium reduction.

In this work, B‐TiO_2_@Co_2_P‐X S‐scheme heterojunction with metal‐oxygen‐hydrogen (M‐O‐H) modification was prepared by hydrothermal method for U(VI) reduction from uranium mine wastewater. Relying on the theoretical calculation and other characterization analysis, we confirmed that M‐O‐H was successfully in situ modified on B‐TiO_2_@Co_2_P‐X to increase the active site for uranium capture, and also confirmed that the introduction of M‐O‐H also increased the adsorption energy of free uranyl ions. The experiments and DFT results showed that the presence of an internal electric field (IEF) in the heterojunction can separate the photo‐charge more efficiently, thus triggering efficient U(VI) reduction. This study provides a novel avenue for the development of an advanced photocatalyst with high‐efficiency photoelectron separation for efficient photo‐assisted uranium reduction from uranium mine wastewater.

## 2 Result and Discussion

### 2.1 Material Synthesis and Characterization

**Figure 1:**
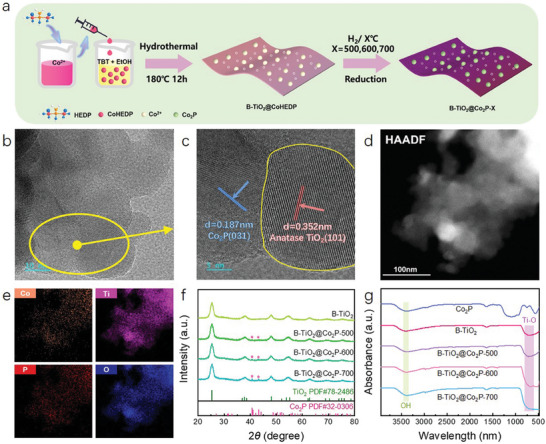
a) Preparation flow chart of B‐TiO_2_@Co_2_P‐500. b) TEM image of B‐TiO_2_@Co_2_P‐500, c,d) HRTEM of B‐TiO_2_@Co_2_P‐500. e) TEM‐EDX elemental mapping images of B‐TiO_2_@Co_2_P‐500. f) XRD pattern of B‐TiO_2_ and B‐TiO_2_@Co_2_P‐X. g) The FTIR spectra of Co_2_P, B‐TiO_2_ and B‐TiO_2_@Co_2_P‐X.

The synthesis of the B‐TiO_2_@Co_2_P‐X hybrid nanosheet was schematically illustrated in **Figure** **1**a. First, the black TiO_2_(B‐TiO_2_) rich in Co_2_P precursors (B‐TiO_2_@CoHEDP) was synthesized by a simple alcohol‐assisted solvothermal method derived from a previously reported work.^[^ ^32^ ^]^ Next, B‐TiO_2_@Co_2_P‐X (X = 500, 600, 700 °C) hybrid nanosheets were further synthesized by calcination at different temperatures with Co_2_P particles supported on the B‐TiO_2_ nanosheets. As shown by the transmission electron microscope (TEM) in Figure 1b, we can observe that the Co_2_P particles with 20–30 nm was uniformly dispersed on the TiO_2_ nanosheet surface. The contact boundary between Co_2_P and B‐TiO_2_ and lattice defects of TiO_2_ by high‐resolution TEM (HRTEM) images were further observed (Figure 1c,d). Figure 1d shows two kinds of clear lattice fringes with interplanar spacing of 0.351 and 0.185 nm, corresponding to the (101) facets of anatase‐TiO_2_ and the (031) facets of orthorhombic‐Co_2_P, respectively. In addition, the lattice defects caused by oxygen vacancies can be seen at the interface of titanium dioxide.^[^ ^33^ ^]^ The Energy dispersive X‐ray (EDX) elemental mapping of B‐TiO_2_@Co_2_P‐500 hybrid nanosheets showed that Ti, O, P, and Co elements were uniformly distributed throughout the selected region, confirming that Co_2_P was successfully synthesized on the B‐TiO_2_ surface (Figure 1e).

The structure of B‐TiO_2_@Co_2_P‐X hybrid nanosheets was analyzed in depth by powder X‐ray diffraction (XRD) and Fourier transform infrared (FT‐IR). As shown in Figure 1f, B‐TiO_2_@Co_2_P‐500 hybrid nanosheets exhibited distinct six broad peaks at 25.2°, 37.9°, 48.1°, 54.1°, 55.2°, and 75.2°, corresponding to (101), (004), (200), (105), (211), and (220) anatase‐TiO_2_ crystal planes, respectively.^[^ ^34^ ^]^ We observed the diffraction peaks of Co_2_P at 40.7°and 43.2°in the XRD of B‐TiO_2_@Co_2_P‐X, providing further evidence for successful loading of Co_2_P. Moreover, an increase in annealing temperature resulted in enhanced visibility of the characteristic diffraction peaks of Co_2_P, indicating that higher temperatures promoted the stability of its crystal structure.

Figure 1g shows the FT‐IR of B‐TiO_2_, Co_2_P, and B‐TiO_2_@Co_2_P‐X. The absorption band at 581, 3402, and 1078 cm^−1^ for B‐TiO_2_@Co_2_P‐500 hybrid nanosheets corresponded to the Ti‐O band and ‐OH group, respectively,^[^ ^35^, ^36^ ^]^ which confirmed the presence of plentiful M‐O‐H on the B‐TiO_2_@Co_2_P‐500 hybrid nanosheets surface.

**Figure 2:**
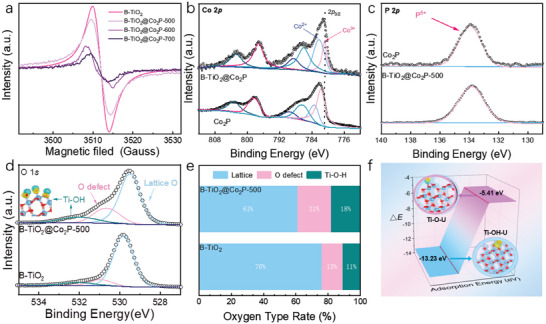
a) ESR signal of oxygen vacancy of B‐TiO_2_, Co_2_P, B‐TiO_2_@Co_2_P‐500, B‐TiO_2_@Co_2_P‐600, B‐TiO_2_@Co_2_P‐700. XPS spectrum of b) Co 2*p*, c) P 2*p*, and d) O 1*s*. e) Oxygen type rate of B‐TiO_2_@Co_2_P‐500 and B‐TiO_2_. (f) Schematic diagram of adsorption energy of Ti‐O and Ti‐O‐H.

To further elucidate the presence of M‐O‐H metallic bond defect in the B‐TiO_2_@Co_2_P‐X hybrid nanosheets, we conducted the electron paramagnetic resonance (ESR) spectrum and X‐ray photoelectron spectroscopy (XPS) measurements. The total XPS spectrum is shown in Figure S1 (Supporting Information). As shown in **Figure** **2**a, the oxygen vacancy signal spectrum of B‐TiO_2_ and B‐TiO_2_@Co_2_P‐X, and B‐TiO_2_@Co_2_P‐X hybrid nanosheets displayed a significantly strong ESR signal, further verifying the existence of abundant oxygen vacancy defect in the B‐TiO_2_@Co_2_P‐X hybrid nanosheets. In the XPS survey spectra of B‐TiO_2_@Co_2_P‐500, the fundamental elements of Ti, O, Co, and P were all displayed (Figure 2; Figure S2, Supporting Information). From the Co 2*p* spectrum (Figure 2b), the peaks at 782.3 ± 0.1 and 797.2 ± 0.1 eV corresponded to Co 2*p*_1/2_ and Co 2*p*_3/2_, respectively, while the peaks at 781.8 ± 0.1, 789.7 ± 0.1, 783.1 ± 0.1, and 786.7 ± 0.1 eV corresponded to Co^3+^, respectively.^[^ ^37^ ^]^ Moreover, the high‐resolution P 2*p* spectrum for Co_2_P and B‐TiO_2_@Co_2_P‐500 all presented an isolated phosphorus peak at 133.8 ± 0.1 eV, further elucidating the successfully synthesized Co_2_P (Figure 2c). In Ti 2*p* XPS spectra, the peaks at ≈458.4 ± 0.1 and ≈464.2 ± 0.1 eV were ascribed to the Ti 2*p*_3/2_ and Ti 2*p*_1/2_ peaks, respectively (Figure S2, Supporting Information).

Figure 2d showed that the peaks of the O 1*s* spectrum at 530.0 ± 0.1, 531.3 ± 0.1, and 532.9 ± 0.1 eV were attributed to lattice O, surface defect O, and surface hydroxyl O, respectively. Based on the integration corresponding peak area in the O 1*s* spectrum, we further calculated the proportion of various surface O species in the obtained samples. Compared with pristine B‐TiO_2_, the proportion of oxygen defects and Ti‐O‐H bond characteristic peaks in B‐TiO_2_@Co_2_P‐500 was relatively large (Figure 2e). Because of the in‐ situ formation of Co_2_P, a large number of crystallographic defects appeared on the TiO_2_ surface, which made the unsaturated coordination Ti in B‐TiO_2_ more likely to adsorb ‐OH in aqueous solution to form Ti‐O‐H metallic bonds.^[^ ^38^ ^]^ The influence of M‐O‐H on the hydrophilicity of the material is further verified through the water contact Angle experiment. As shown in Figure S10 (Supporting Information), the contact angle of B‐TiO_2_ (48°) is significantly greater than that of B‐TiO_2_@Co_2_P‐500 (29°). According to the rate of oxygen types in Figure 2e, the Ti‐O‐H bond of B‐TiO_2_@Co_2_P‐500 is more than that of B‐TiO_2_. Therefore, we conclude that M‐O‐H can enhance the hydrophilicity of the material. The M‐O‐H metallic bond not only improved the hydrophilicity of the B‐TiO_2_@Co_2_P‐X hybrid nanosheets but also enhanced the specific recognition of U (VI).^[^ ^39^ ^]^ To further validate the effective confinement of uranium precursors by M‐O‐H metallic bond, we employed the density functional theory (DFT) calculations. First, we constructed the TiO_2_ slabs with and without M‐O‐H metallic bond to compare the adsorption energy for the uranyl ion. As shown in Figure 2f, the adsorption energy (*E*_ads_) of uranyl ions on TiO_2_ slabs with M‐O‐H metallic bond were calculated to be −13.23 eV, which was much lower than that of *E*_ads_ of uranyl ions on TiO_2_ slabs without M‐O‐H metallic bond (−5.41 eV), further confirming the specific recognition effect of M‐O‐H metallic bond.

### 2.2 Optical and Electrochemical Characteristics of B‐TiO_2_@Co_2_P‐X

**Figure 3:**
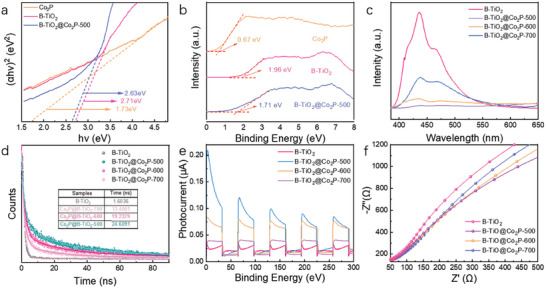
a) Tauc/Davis ‐Mott plots, showing (αh*v*)^1/2^ versus h*v* curves of Co_2_P, B‐TiO_2_ and B‐TiO_2_@Co_2_P‐500. b) Valence band edge map of Co_2_P, B‐TiO_2_ and, B‐TiO_2_@Co_2_P‐500. c) PL spectra of Co_2_P, B‐TiO_2_, and B‐TiO_2_@Co_2_P‐X. d) Time‐resolved PL spectra of B‐TiO_2_ and B‐TiO_2_@Co_2_P‐X. e) Transient photocurrent responses of B‐TiO_2_ and B‐TiO_2_@Co_2_P‐X. f) Impedance spectrum of B‐TiO_2_ and B‐TiO_2_@Co_2_P‐X.

The changes in the energy band structure caused by the hybridization of Co_2_P and B‐TiO_2_ were further analyzed by optical means. Figure S3 (Supporting Information) and **Figure** **3**a showed the UV–vis absorption spectra and band gap profiles of Co_2_P, B‐TiO_2_, and B‐TiO_2_@Co_2_P‐500. Due to the UV‐vis DRS, the band gap (*E*_g_) of Co_2_P, B‐TiO_2_, and B‐TiO_2_@Co_2_P‐500 was further calculated based on the Tauc/Davis–Mott model. As shown in Figure 3a, the *E*_g_ values for Co_2_P, B‐TiO_2_, and B‐TiO_2_@Co_2_P‐500 were estimated as 1.73, 2.71, and 2.63 eV, respectively. Figure 3b presents the valence band (VB) edge spectrum of Co_2_P and B‐TiO_2_. The positions of VB edges for Co_2_P and B‐TiO_2_ were located at 0.67, 1.96, and 1.71 eV, respectively. Considering the *E*_g_ value, the conduction band (CB) edge positions were calculated to be −1.06, −0.75, and −0.89 eV for Co_2_P, B‐TiO_2_, and B‐TiO_2_@Co_2_P‐500, respectively. According to the above analysis, we further established the band‐level diagram of pristine Co_2_P, B‐TiO_2_, and B‐TiO_2_@Co_2_P‐500. (Figure S4, Supporting Information).

Figure 3c displays the photoluminescence (PL) spectra of the as‐prepared B‐TiO_2_ and B‐TiO_2_@Co_2_P‐X. Compared with pristine B‐TiO_2_, B‐TiO_2_@Co_2_P‐X all presented lower PL intensities, which indicated that the hybridization of B‐TiO_2_ with Co_2_P effectively enhanced the electron‐hole separation efficiency. The above results were further demonstrated by time‐resolved photoluminescence spectroscopy (TRPL) (Figure 3d). The kinetics of all samples in TRPL conformed to the second‐order exponential decay behavior. As displayed in Figure 3d, the weighted mean lifetimes of photogenerated electrons (τ_1_) for B‐TiO_2_@Co_2_P‐X were 24.61 ns, 13.40 ns, and 19.23 ns, respectively, which were significantly longer than that of pure B‐TiO_2_ (1.60 ns), indicating that functional modification of Co_2_P nanoparticles on pristine TiO_2_ nanosheets surface can reduce the probability of photo‐induced electron deactivation, thereby enhancing the photocatalytic activity.

As shown by i–t curves, the B‐TiO_2_@Co_2_P‐500 (0.21 µA cm^−2^) exhibited a much higher photo–current density than that of pure B‐TiO_2_ (0.03 µA cm^−2^), proclaiming that B‐TiO_2_@Co_2_P‐X generated more photo‐induced electrons for the photocatalytic uranium reduction during the photocatalytic reaction process (Figure 3e). Meanwhile, the EIS curves displayed that the arc radius of B‐TiO_2_@Co_2_P‐X was much smaller than that of pristine TiO_2_ nanosheets, indicating better interfacial charge transfer kinetics between Co_2_P and TiO_2_, which was conducive to supporting the results of photocurrent measurements (Figure 3f).^[^ ^40^ ^]^ Taken together, these results showed that constructing S‐scheme heterojunction by functionalizing Co_2_P nanoparticles on the TiO_2_ nanosheet surface effectively promotes electron‐hole separation efficiency and extends electron lifetime, thereby promoting the photocatalytic reduction of uranium.

### 2.3 Evaluation of U(VI) Reduction Performance

**Figure 4:**
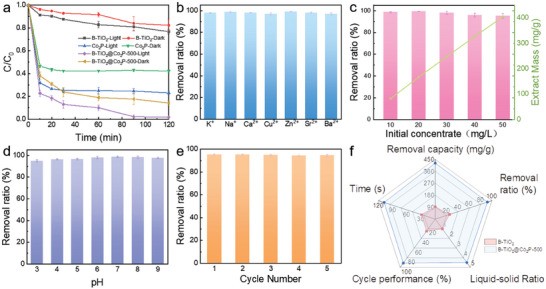
a) Reaction kinetics of the sample under irradiation and non‐irradiation. b) The removal ratio of U(VI) under the influence of different interfering ions by B‐TiO_2_@Co_2_P‐500. c) Effect of raw U(VI) concentration on U(VI) removal and the capacity of uranium by B‐TiO_2_@Co_2_P‐500. d) Effect of B‐TiO_2_@Co_2_P‐500 in different pH on U(VI) removal ratio. e) Cyclic stability test of B‐TiO_2_@Co_2_P‐500. f) Comprehensive performance evaluation of B‐TiO_2_ and B‐TiO_2_@Co_2_P‐500.

The M‐O‐H‐enriched B‐TiO_2_@Co_2_P‐X provided a hopeful platform for photo‐assisted uranium reduction from uranium wastewater. Through photocatalytic experiments on B‐TiO_2_@Co_2_P‐X, B‐TiO_2_@Co_2_P‐500 showed the best uranium removal ratio (Figure S5, Supporting Information). Therefore, B‐TiO_2_@Co_2_P‐500 was taken as an example, we further evaluated the ability to remove uranium.^[^ ^41^ ^]^ **Figure** **4**a shows the reaction time curves of Co_2_P, B‐TiO_2,_ and B‐TiO_2_@Co_2_P‐500 to U(VI) under simulated sunlight irradiation and non‐irradiation conditions. To better demonstrate the advantages of B‐TiO_2_@Co_2_P‐500 reduced uranium, we have respectively drawn the uranium absorption ratio (Figure S7a,b, Supporting Information) of the three samples before and after irradiation and attached the bar graph (Figure S7c,d, Supporting Information) of the final reduction efficiency. In the dark conditions, the B‐TiO_2_@Co_2_P‐500 hybrid nanosheets exhibited 4.5 times larger removal capacity than pristine B‐TiO_2_ nanosheets, which was ascribed to the presence of M‐O‐H metallic bond on B‐TiO_2_@Co_2_P‐500 hybrid nanosheets, serving as the confinement capture sites for free uranyl ions on the uranium solution. After the introduction of the simulated sunlight, all as‐prepared samples displayed a significantly enhanced removal ability for U(VI). The B‐TiO_2_@Co_2_P‐500 presented a promoted removal ability of 98% in 90 min, which was a great improvement compared with that by pristine B‐TiO_2_ (21% in 120 min) and Co_2_P (73% in 120 min). The aforementioned results validate the synergistic effect of the S‐scheme heterojunction and M‐O‐H integration, which facilitates efficient photoelectron transfer and provides abundant active sites, thereby accelerating the reaction kinetics of U(VI).

To better illustrate the outstanding photocatalytic uranium of B‐TiO_2_@Co_2_P‐500, we have conducted a comprehensive comparison with numerous reported photocatalysts. As depicted in Table S1 (Supporting Information), B‐TiO_2_@Co_2_P‐500 exhibits superior catalytic efficiency compared to the aforementioned catalysts. Furthermore, we compared the performance of B‐TiO_2_@Co_2_P‐500 with that of commercially available photocatalysts P25‐TiO_2_ and g‐C_3_N_4_. As illustrated in Figure S11 (Supporting Information), after 120 minutes of light‐induced reaction, P25‐TiO_2_ and g‐C_3_N_4_ achieved uranium removal ratios of 63% and 52%, respectively. Conversely, within just 30 minutes under light irradiation, B‐TiO_2_@Co_2_P‐500 accomplished a remarkable uranium reduction rate of 90%. Evidently, B‐TiO_2_@Co_2_P‐500 demonstrates exceptional catalytic performance.

The most suitable solid–liquid ratio was determined by testing the ratio of different materials to uranium solution (Figure S8, Supporting Information). The composition of uranium mine wastewater was complex and contained a variety of metal cations. Metal cations will compete with uranium for catalytic active sites, which had a great influence on the removal effect of U(VI). As a consequence, we further evaluated the anti‐interference and stability of B‐TiO_2_@Co_2_P‐500. At first, we evaluated the uranium removal ability of B‐TiO_2_@Co_2_P‐500 under the competitive interference of a large number of metal cations (K^+^, Na^+^, Ca^2+^, Mg^2+^, etc.). As shown in Figure 4b, the B‐TiO_2_@Co_2_P‐500 still be kept > 95% U(VI) removal ratio under the competition and interference of different metal cations. We further assessed the efficacy of B‐TiO_2_@Co_2_P‐500 in removing and reducing uranium at varying U(VI) concentrations, while also investigating its capacity for uranium enrichment (Figure 4c). Specifically, B‐TiO_2_@Co_2_P‐500 exhibited a consistently high level of over 90% reduction efficiency for U(VI) when initial concentrations ranged from 10 to 50 ppm. Notably, at an initial concentration of 50 mg L^−1^, B‐TiO_2_@Co_2_P‐500 achieved a remarkable uranium reduction capacity of up to 421.96 mg g^−1^ under simulated sunlight irradiation. Figure 4d showed the B‐TiO_2_@Co_2_P‐500 still maintained a high U(VI) removal capacity (removal rate > 90%) in a wide pH range of 3–9. Furthermore, the chemical stability and ability of cycling via the B‐TiO_2_@Co_2_P‐500 were also verified. After five cycles, the removal rate of U (VI) by B‐TiO_2_@Co_2_P‐500 remained at a relatively high level of over 90% (Figure 4e).^[^ ^42^ ^]^ The above results verified that the B‐TiO_2_@Co_2_P‐500 served as one of the ideal catalyst candidates for the efficient reduction of uranium from complex uranium mine wastewater environments.

### 2.4 DFT Simulation of Electron Transfer in S‐scheme Heterojunction

**Figure 5:**
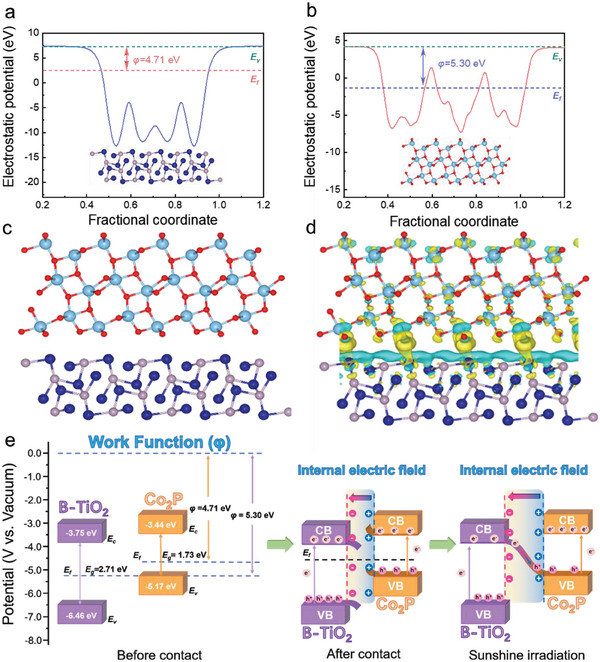
a) The calculation results of the Co_2_P work function. b) The calculation results of the TiO_2_ work function. c) Contact model of B‐TiO_2_ and Co_2_P. d) Electron transfer model of B‐TiO_2_@Co_2_P‐X. e) Schematic diagram of electron transfer principle of S‐scheme heterojunction.

Density functional theory (DFT) calculations were used as an effective means to reveal the interfacial electron transfer pathway and formation mechanism in the B‐TiO_2_@Co_2_P‐500 heterojunction. As shown in **Figure** **5**a,b, the charge density difference distribution of B‐TiO_2_@Co_2_P‐500 presented the charge redistribution close to the heterojunction interface of B‐TiO_2_@Co_2_P‐500. The accumulation of net charges led to the formation of a huge internal electric field (IEF) at the interface of B‐TiO_2_@Co_2_P‐500, with an electric field direction from Co_2_P to B‐TiO_2_. The driving force of the S‐scheme transfer mechanism on B‐TiO_2_@Co_2_P‐500 was further revealed by work function (Φ) calculations. The Fermi levels (*E*_f_) of predominantly exposed TiO_2_ (101) and Co_2_P (031) surfaces were calculated based on the equation of *E*_f_ ‐ *E*_vac_ = Φ, in which the *E*_vac_ is the energy of vacuum level (assumed as 0 eV). As shown in Figure 5c,d, the Φ of the TiO_2_ (101) and Co_2_P (031) planes was estimated as 5.30 and 4.71 eV versus vacuum level, respectively. Accordingly, *E*_f_ for the TiO_2_ (101) and Co_2_P (031) was calculated to be −1.09 and 2.65 eV, respectively. The *E*_f_ of Co_2_P (031) was more positive than that of TiO_2_ (101). After close contact between the TiO_2_ (101) and Co_2_P (031), the photo‐excited electrons tended to transfer from the Co_2_P (031) to TiO_2_ (101), thus achieving *E*_f_ equilibrium. As shown in Figure 5e, the area near the B‐TiO_2_@Co_2_P‐X interface was charged, resulting in a huge IEF. This IEF was conducive to the directional migration of charge carriers by the S‐scheme transfer pathway, which depletes photoelectrons on Co_2_P (031) and holes on TiO_2_ (101). The spatially separated photoelectrons on Co_2_P (higher CB) and holes on TiO_2_ (lower VB) possess stronger redox capabilities and longer lifetimes than either Co_2_P or TiO_2_.^[^ ^43^, ^44^, ^45^, ^46^ ^]^

**Figure 6:**
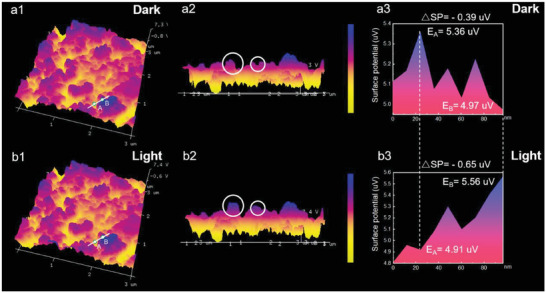
a1) Surface potential distribution diagram under dark conditions. a2) Transverse potential distribution diagram under dark conditions. a3) SPV spectra under dark conditions. b1) Surface potential distribution diagram under illumination conditions. b2) Transverse potential distribution diagram under illumination conditions. b3) SPV spectra under illumination conditions.

To further verify the photo‐induced charge separation and transfer direction at the B‐TiO_2_@Co_2_P‐X interface, the spatial distribution of charges was studied by the in situ Kelvin probe force microscope (KPFM).^[^ ^47^, ^48^, ^49^, ^50^, ^51^, ^52^ ^]^ The surface photovoltage of B‐TiO_2_@Co_2_P‐500 under dark and light conditions was compared by atomic force microscope (AFM) (**Figure** **6**). Figure 6a1,b1 showed the overall distribution of surface potential under dark and light conditions, respectively. For the details in the 3D AFM image (Figure 6a2,b2), the white mark was the area where the surface potential increased obviously from darkness to illumination. As shown in the surface photovoltage spectrum (SPV) (Figure 6a3,b3), the surface potential of B‐TiO_2_@Co_2_P‐500 at position A decreased by ≈ 0.45 µV, while the surface potential of position B increased by ≈ 0.59 µV during the transition from dark to bright, indicating that the process of electron transfer from position B to position A was generated by introducing illumination, which confirmed the existence of photogenerated electron transfer powered by the interfacial electric field on B‐TiO_2_@Co_2_P‐500.^[^ ^53^, ^54^, ^55^, ^56^ ^]^

### 2.5 Reaction Mechanism of Photocatalytic Reduction of Uranium

**Figure 7:**
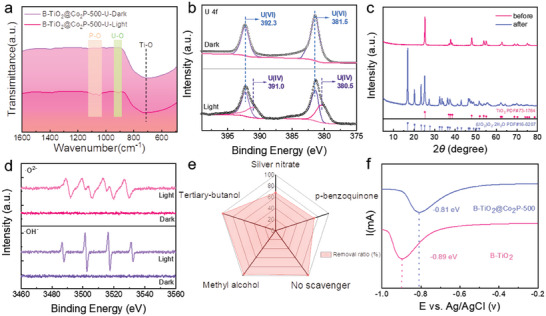
a) FITR spectrum of B‐TiO_2_@Co_2_P‐500‐U‐Dark and B‐TiO_2_@Co_2_P‐500‐U‐Light. b) XPS spectrum of U 4*f* before and after photoreaction. c) XRD spectrum of before and after photoreaction. d) The ESR signals of ·O_2_^−^ and ─OH^−^ before and after illumination. e) Radar chart of experimental results of the scavenger. (f) Reduced LSV curve of U(VI).

To determine the present state of U(VI) on the catalyst surface after photo‐reduction and further clarify the reaction mechanism of the photocatalytic U(VI) reduction process, the reacted products were systematically identified. The B‐TiO_2_@Co_2_P‐500 after reacting under dark and light conditions were further collected and named B‐TiO_2_@Co_2_P‐500‐U‐Dark and B‐TiO_2_@Co_2_P‐500‐U‐Light, respectively. The B‐TiO_2_@Co_2_P‐500‐U‐Dark after the photocatalytic reaction was tested by FTIR (**Figure** **7**a). Compared with B‐TiO_2_@Co_2_P‐500‐U‐Dark, a new peak appeared at 920 cm^−1^ corresponding to the U─O bond, further confirming the presence of uranium species.^[^ ^57^ ^]^ As shown in Figure 7b, the U 4*f* signals were presented in XPS spectra of B‐TiO_2_@Co_2_P‐500‐U‐Dark and B‐TiO_2_@Co_2_P‐500‐U‐Light. For B‐TiO_2_@Co_2_P‐500‐U‐Dark, the distinctive peaks located at 392.9 ± 0.1 and 382.1 ± 0.1 eV were exclusive to U 4*f*_5/2_ and U 4*f*_7/2_ of U(VI) respectively, which powerfully demonstrated that UO_2_^2+^ was indeed selectively adsorbed by M‐O‐H. Compared with the B‐TiO_2_@Co_2_P‐U‐Dark, the U 4*f* spectra of B‐TiO_2_@Co_2_P‐500‐U‐Light presented two additional characteristic peaks at 391.5 ± 0.1 and 380.7 ± 0.1 eV are classified as U 4*f*_5/2_ and U 4*f*_7/2_ of U(IV), respectively, indicating that the part of enriched uranium was reduced by the photocatalytic process. Furthermore, the linear scanning voltammetry (LSV) curve presented a significantly reduction peak, further confirming the reduction potential of uranium could be reduced by M‐O‐H. In addition, the samples were tested after the photocatalytic reaction by XRD measurement. The XRD spectrum of B‐TiO_2_@Co_2_P‐500‐U‐Light presented that the uranium species mainly existed in the form of uranium oxide hydrate ((UO_2_) O_2_⋅2H_2_O)) (Figure 7c).

To determine whether other species with redox ability were involved in the reaction during the photocatalytic uranium reduction reaction, thereby driving the production of (UO_2_)O_2_·2H_2_O, We further analyzed the species with redox ability during the photocatalytic reduction of U(VI) by ESR (Figure 7d). Before the light reaction, no signal was generated. While after introducing light irradiation, the ESR spectra displayed corresponding signals of superoxide radical (·O_2_^−^) and hydroxyl radical (·OH^−^), respectively, which undoubtedly confirmed our conjecture that the presence of ·O_2_^−^ and ·OH^−^ in the photocatalytic reduction of U(VI) was likely to be the main promoter of (UO_2_)O_2_·2H_2_O production reason. To further clarify the main reason for the production of (UO_2_)O_2_·2H_2_O, we conducted scavenger experiments to verify. Under the same conditions as all photocatalytic reactions, tert‐butyl alcohol, methanol, *p*‐benzoquinone, and Silver nitrate were added to selectively eliminate ·OH^−^, h^+^(holes), ·O_2_^−^, and e^−^(electrons generated), respectively. The results of the photocatalytic reduction of U(VI) experiments showed (Figure 7e; Figure S9, Supporting Information) that the addition of silver nitrate, and *p*‐benzoquinone directly affected the photocatalytic reduction ability of the catalyst for U(VI), further proving that ·O_2_^−^ and e^−^ were the main active substances in the photocatalytic reduction of U(VI).^[^ ^58^ ^]^ In addition, the reaction system with the addition of methanol accelerated the separation of electron‐hole pairs due to the elimination of h^+^ by methanol, thereby improving the photocatalytic reduction rate. Notably, the reaction system with the addition of tert‐butanol had a significant increase in both photocatalytic rate and removal ratio, which was attributed to the reduction of ·OH^−^, and the removal of ·OH^−^ prevents the secondary oxidation of U(VI) during the reaction process.

**Figure 8:**
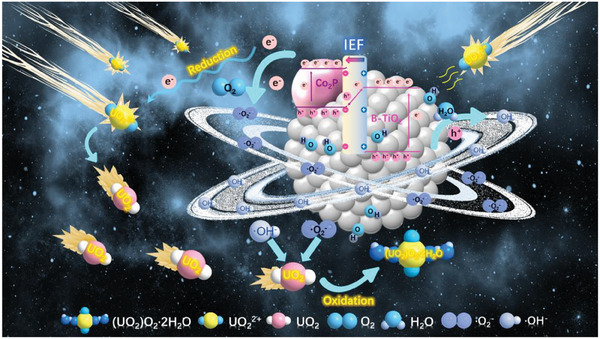
Reaction mechanism diagram of photocatalytic reduction of uranium by B‐TiO_2_@Co_2_P‐500.

With the above results, we condensed the reaction mechanism of B‐TiO_2_@Co_2_P‐500 photocatalytic reduction of uranium (**Figure** **8**) and listed the reaction equation. When the catalyst is photoexcited (Equation 1), part of the e^−^ will directly reduce with U(VI) through Equation 2, and the other part of e^−^ will combine with O_2_ to form ·O_2_^−^ by Equation 3, and the h^+^ will combine with water to form ·OH^−^ by Equation 4. Under the synergic action of ·O_2_^−^ and ·OH^−^, the U(IV) on the surface of the B‐TiO_2_@Co_2_P‐500 nano‐hybrid sheet is further oxidized to (UO_2_)O_2_·2H_2_O by Equation 5.

(1) $$\text{B-Ti}O_{2}@Co_{2}P+\mathrm{hv}\to h^{+}+e^{-}$$

(2) $$U\left(\mathrm{VI}\right)+e^{-}\to U\left(\mathrm{IV}\right)$$

(3) $$e^{-}+O_{2}\to \cdot O_{2}^{-}$$

(4) $$h^{+}+H_{2}O\to \cdot {\mathrm{OH}}^{-}+h^{+}$$

(5) $$U\left(\mathrm{IV}\right)+\cdot OH^{-}+\cdot O_{2}^{-}\to \left({\mathrm{UO}}_{2}\right)O_{2}\cdot 2H_{2}O$$

Based on the analysis of charge transfer characteristics and theoretical calculations, a mechanism for synergistic enhancement of photo‐assisted uranium reduction by S‐scheme heterojunction and surface M‐O‐H dangling bond was proposed (Figure 8). With the aid of light excitation, the charge carriers generated by B‐TiO_2_@Co_2_P‐500 migrate directionally under the internal electric field and interface band bending, achieving effective electron‐hole separation in S‐scheme heterojunctions. The S‐scheme charge migration phenomenon maximizes the retention of B‐TiO_2_@Co_2_P‐500 redox ability. In addition, the M‐O‐H metallic bonding on the B‐TiO_2_@Co_2_P‐500 surface provided a large number of active sites for efficient and selective capture of uranium, thus accelerating the adsorption kinetics of uranium. It is worth noting that electrons were also key to photocatalytic uranium reduction. As the main force in the process of uranium oxidation‐reduction crystallization, ·OH^−^ and ·O_2_^−^ contribute to the formation of (UO_2_)O_2_·2H_2_O.

## 3 Conclusion

In conclusion, B‐TiO_2_@Co_2_P‐500 S‐scheme heterojunction with M‐O‐H metallic bond was successfully prepared for U(VI) reduction from uranium mine wastewater. The M‐O‐H metallic bond not only enhanced the hydrophilicity of the B‐TiO_2_@Co_2_P‐500 heterojunction but also improved their specific recognition ability toward U(VI). Meanwhile, the B‐TiO_2_@Co_2_P‐500 heterojunction exhibited superior photoelectron separation efficiency during U(VI) reduction, which was conducive to further enhancing its catalytic performance in U(VI) reduction. This study provides a novel avenue for the development of an advanced photocatalyst with high‐efficiency photoelectron separation for efficient photo‐assisted uranium reduction from uranium mine wastewater.

## 4 Experimental Section

### Chemical Materials

Cobalt nitrate hexahydrate (CoNO_3_·6H_2_O), Etidronic acid (C_2_H_8_O_7_P_2_), Titanium butoxide (C_16_H_36_O_4_Ti), Ethanol (CH_3_CH_2_OH), tert‐Butanol (C_4_H_10_O), Methanol (CH_3_OH), *p*‐Benzoquinone (C_6_H_4_O_2_), Silver nitrate (AgNO_3_). The above drugs were purchased from Aladdin Biochemical Technology (Shanghai, China). The ultrapure water (UPW) used in the study was resistivity 18.25 mω·cn. All drugs were used directly without purification before use.

### Synthesis of the B‐TiO_2_@Co_2_P‐X

First of all, 0.2 mm of cobalt nitrate hexahydrate (CoNO_3_‐6H_2_O) and 0.1 mm of hydroxyethylenediphosphonic acid (HEDP) were fully dissolved in 10 mL of ultrapure water, and the pH value of the fully dissolved solution was adjusted to 5 and stirred for 1 h to obtain a pink‐purple cobalt phosphonate emulsion. Then prepare tetra butyl titanate solution, add 4 mL of tetra butyl titanate (TBT) to 40 mL of alcohol, and then stir vigorously at low temperature, and slowly drop in cobalt phosphonate emulsion. The well‐mixed solution was loaded into an autoclave with a polytetrafluoroethylene (PTFE) liner and held at 180 °C for 12 h. After the autoclave was cooled to room temperature, the solution in the liner was repeatedly washed alternately with ultrapure water and ethanol and dried in a vacuum drying oven at 60 °C. The obtained material was named B‐TiO_2_@CoHEDP. Finally, the obtained B‐TiO_2_@CoHEDP was annealed at 600 °C under an H_2_ atmosphere for 2 h. We prepared a series of samples annealed at different temperatures for comparison, named B‐TiO_2_@Co_2_P‐X. X = 500, 600, and 700 °C.

### Synthesis of B‐TiO_2_

The preparation was the same as that of B‐TiO_2_@Co_2_P‐X, except that cobalt phosphonate emulsion was not added.

### Density Functional Theory

The work function and adsorption energy of TiO_2_ and Co_2_P are calculated by density functional theory (DFT), and the model of electron transfer after interface contact between TiO_2_ and Co_2_P is simulated.

### Material Characterization and Performance Evaluation

Methods for evaluating the properties of materials and characterization instruments are presented in Supporting information.

## Conflict of Interest

The authors declare no conflict of interest.

## Supporting information

Supporting Information

## Data Availability

Data sharing is not applicable to this article as no new data were created or analyzed in this study.
