# Supplementary material for: In Situ Metal‐Oxygen‐Hydrogen Modified B‐Tio2@Co2P‐X S‐Scheme Heterojunction Effectively Enhanced Charge Separation for Photo‐assisted Uranium Reduction
Source: Adv Sci (Weinh). 2023 Dec 4;11(5):2305439. doi: 10.1002/advs.202305439 (PMC10953717; doi:10.1002/advs.202305439)
Supplement: Supplementary file 1 — Supporting Information [file ADVS-11-2305439-s001.pdf]

ADVANCED SCIENCE
Open Access

Supporting Information

for *Adv. Sci.*, DOI 10.1002/advs.202305439

In Situ Metal-Oxygen-Hydrogen Modified B-Tio_2@Co_2P-X S-Scheme Heterojunction Effectively Enhanced Charge Separation for Photo-assisted Uranium Reduction

Fucheng Zhang, Huanhuan Dong, Yi Li, Dengjiang Fu, Lu Yang, Yupeng Shang, Qiuyang Li, Yuwen Shao, Wu Gang, Tao Ding, Tao Chen and Wenkun Zhu**

Supporting information

In-situ Metal-oxygen-hydrogen Modified B-TiO_2@Co_2P-X S-scheme Heterojunction Effectively Enhanced Charge Separation for Photo-assisted Uranium Reduction

*Fucheng Zhang [a#], Huanhuan Dong [a#], Yi Li [b#], Dengjiang Fu [a], Lu Yang [a], Yupeng Shang [a], Qiuyang Li [a], Yuwen Shao [a], Wu Gang [a], Tao Ding [c], Tao Chen [*a], Wenkun Zhu [*a]*

[a]State Key Laboratory of Environment-friendly Energy Materials, National Co-innovation Center for Nuclear Waste Disposal and Environmental Safety, Sichuan Co-Innovation Center for New Energetic Materials, Nuclear Waste and Environmental Safety Key Laboratory of Defense, School of National Academy of Defense Technology, Southwest University of Science and Technology, Mianyang, Sichuan 621010, People's Republic of China.

[b]School of materials and energy, University of Electronic Science and Technology, Chengdu, 610000, China.

[c]University of Science and Technology of China, National Synchrotron Radiation Laboratory, Hefei 230029, People's Republic of China.

# These authors contributed equally to this work.

*Corresponding author: chent@swust.edu.cn (*T. Chen*); zhuwenkun@swust.edu.cn (W. Zhu)

1.Material Characterization and Performance Evaluation

1.1 U(VI) Photoreduction Tests

All photocatalytic experiments were carried out using 5 mg photocatalyst and 20 ml U(VI) solution [$C_{U(VI)}$ = 8 mg/L, 10 mg/L, 20 mg/L, 30 mg/L, 40 mg/L, 50 mg/L]. Unless otherwise specified, the pH value of U(VI) solution was adjusted to 5.0. As for the ion interference test, 8 mg/L U(VI) solution with 80 mg/L interference ions was used, and 8 mg/L U(VI) solution was used for the uranium solution, and the ratio was the molar ratio. The solid-liquid ratio in the cyclic test is 1:4. For a photocatalytic experiment, the simulated light source is a 300 W xenon lamp (BL-GHX V, China) with an AM 1.5 G filter. Xenon lamp with full spectrum wavelength and intensity of 200 mW/cm^2 was used to irradiate the reaction system. Within 120 minutes of illumination, samples were taken at 10, 20, 30, 60, 90, and 120 respectively, and the same operation was performed in the dark as a comparison. Adjust the pH of the uranium solution with HCl and NaOH to determine the stability in the pH study. Semiconductor anti-interference ability (CU(VI)). C_M = 1: 1， M = K^+, Na^+, Ca^{2+}, Cu^{2+}, Sr^{2+}, Zn^{2+}, Ba^{2+}). After the photocatalytic reaction, the concentration of UO_2^{2+} was measured by a spectrophotometer at the wavelength of 651.8 nm. Use the following equation to calculate the removal efficiency of U(VI) after photocatalysis:

$$\text{removal efficiency} = (C_0\text{-}C_t)/C_0\times 100\%$$

Where C_0 is the initial concentration of U(VI) and C_t is the concentration of U(VI) after a certain time of reaction.

1.2 Characterization instrument

The morphology of B-TiO_2@Co_2P was obtained by transmission electron microscope (TEM, FEI Talos F200S), high resolution transmission electron microscopy (HRTEM), and scanning electron microscope (SEM, ZEISS Gemini 300). The structure of B-TiO_2@Co_2P was obtained by X-ray diffraction (XRD, Smart Lab). XPS spectrum was recorded by a thermal expandable 20Xi photoelectron spectrometer and monochromatic AI ray source (XPS, Thermo Scientific K-Alpha). The functional groups and chemical bonds of B-TiO_2@Co_2P were obtained by FT-IR (Spectrum One Autoima). The ultraviolet-visible spectrum was analyzed by UV-vis (TU-1901). The surface potential of the catalyst under dark and light conditions was studied by atomic force microscope (AFM, Bruker Dimension ICON). The reaction products were analyzed by electron paramagnetic

resonance (ESR, A300 -10/12). The electrochemical experiment was measured by an electrochemical workstation (CHI660e) in a standard three-electrode system. The Xenon lamp of MICROSOLAR300 model was used in the photocatalytic experiment.

1.3 DFT Calculation Parameters

Density functional Theory (DFT) calculations are performed using the Vienna ab initio simulation package (VASP). The generalized gradient approximation was used with the Perdew-Burke-Ernzerhof (PBE) exchange-correlation density functional. Projector augmented wave (PAW) potentials was utilized to describe the interaction of ionic cores and electrons, and the cut-off energy for the plane-wave basis set was 480 eV. The force convergence criterion used for the geometry relaxation was 0.02 eV Å–1. To decipher the long-range dispersion interactions between the adsorbates and catalysts, the D3 correction method by Grimme et al was employed. Brillouin zone integration was accomplished using Monkhorst–Pack k-point mesh. 1x1x1for geometry optimization and 7x2x1for electronic structure computations. In all model calculations, A vacuum of at least 15 A is used along the Z-axis.

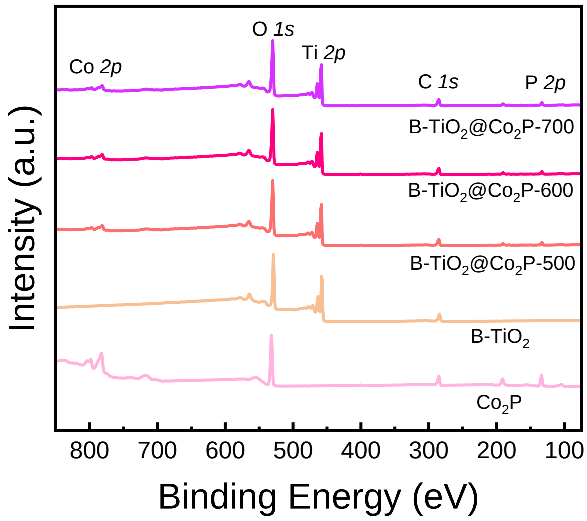

Figure S1. The total XPS spectrum of B-TiO_2, Co_2P and B-TiO_2@Co_2P-X.

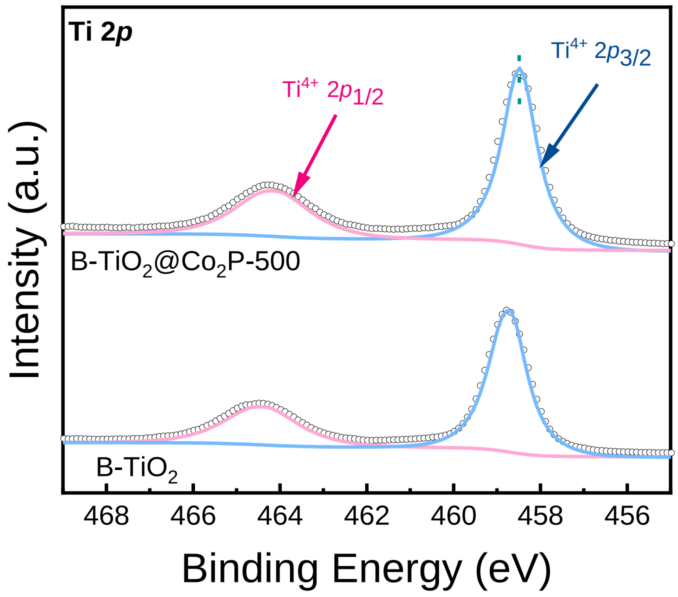

Figure S2. The XPS spectra of Ti 2*p*

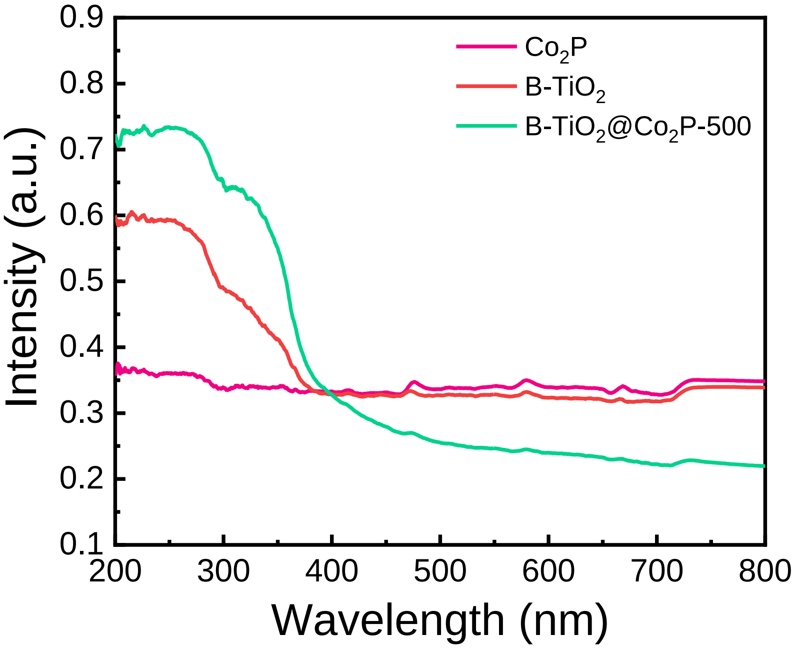

Figure S3. UV-Vis absorption spectra of B-TiO_2, Co_2P and B-TiO_2@Co_2P-500.

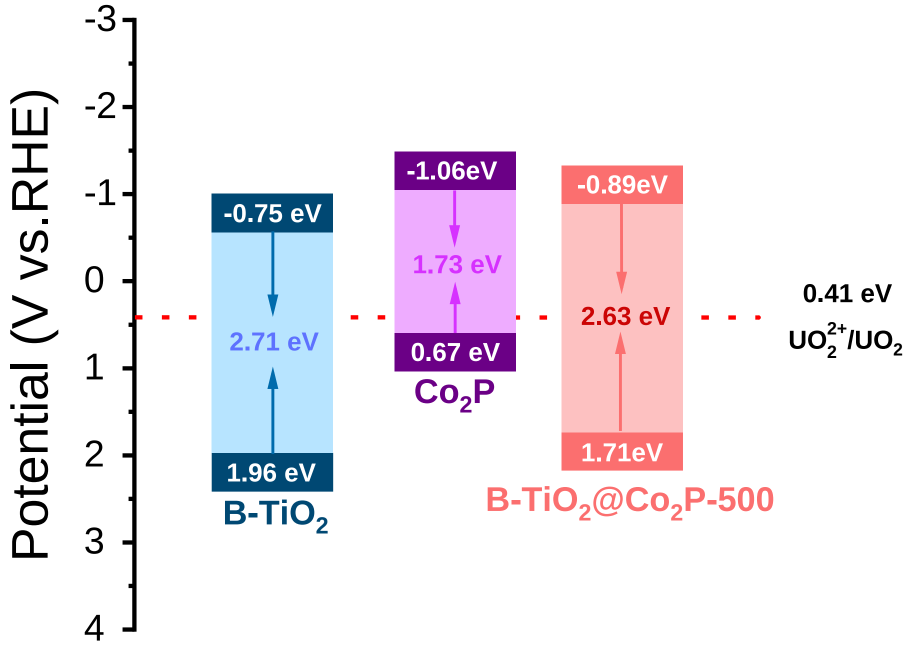

Figure S4. The band level diagram of pristine Co_2P, B-TiO_2 and B-TiO_2@Co_2P-500.

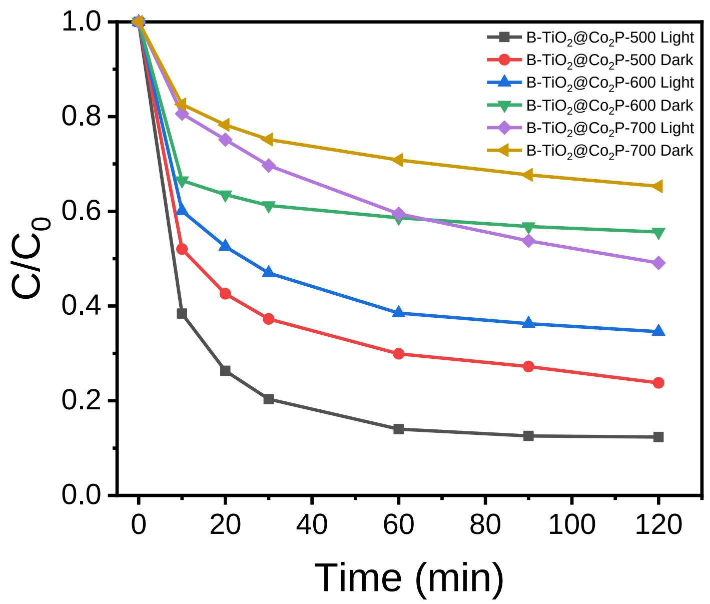

Figure S5. The reaction time curves of B-TiO_2@Co_2P-500, B-TiO_2@Co_2P-600 and B-TiO_2@Co_2P-700.

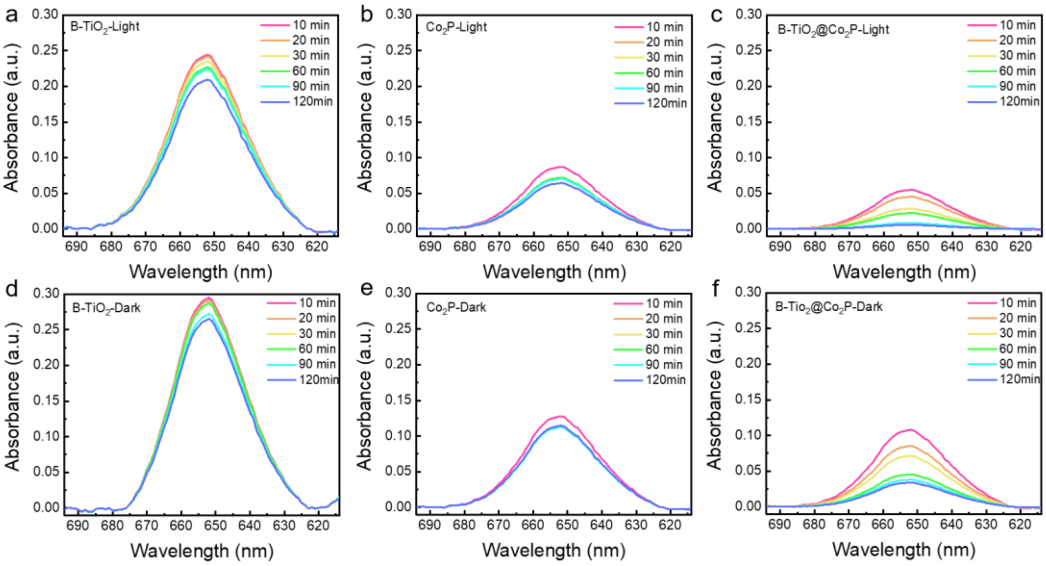

Figure S6. (a-c) The absorbance of U(VI) without irradiation for B-TiO_2, Co_2P, and B-TiO_2@Co_2P-500. (d-e) The absorbance of U(VI) with irradiation for B-TiO_2, Co_2P, and B-TiO_2@Co_2P-500.

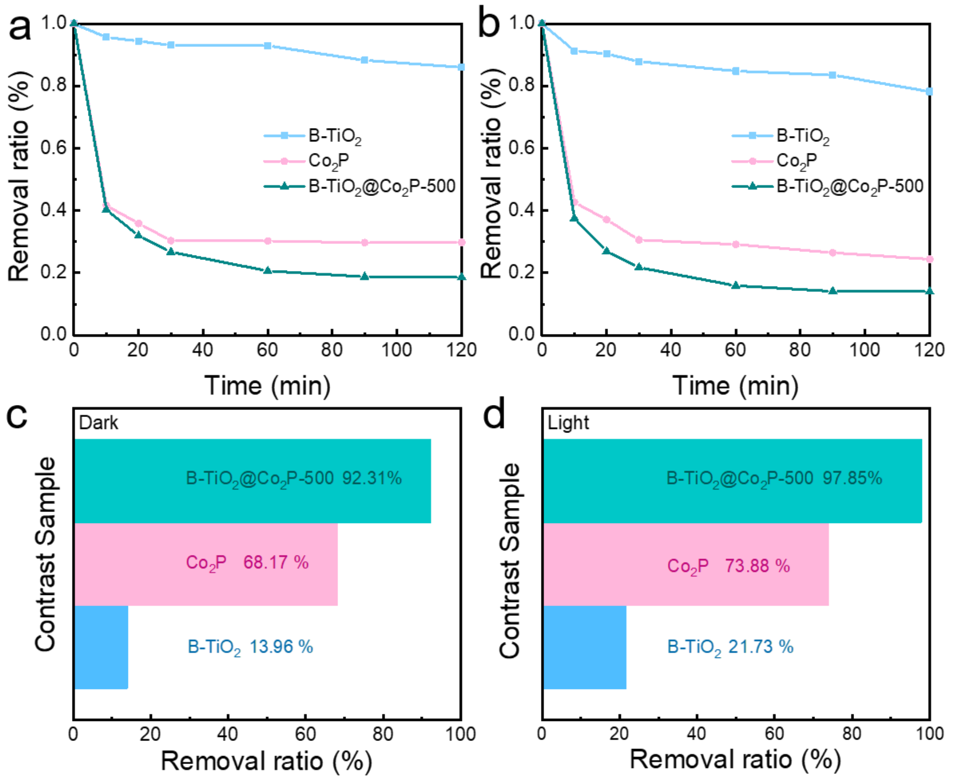

Figure S7. (a) Uranium reduction time curve of B-TiO_2, Co_2P and B-TiO_2@Co_2P-500 under non-illumination condition (b) Uranium reduction time curve of B-TiO_2,Co_2P and B-TiO_2@Co_2P-500 under illumination condition (c) B-TiO_2, Co_2P and B-TiO_2@Co_2P under non-illumination condition P-500 final uranium reduction efficiency (d) Final uranium reduction efficiency of B-TiO_2, Co_2P and B-TiO_2@Co_2P-500 under light conditions.

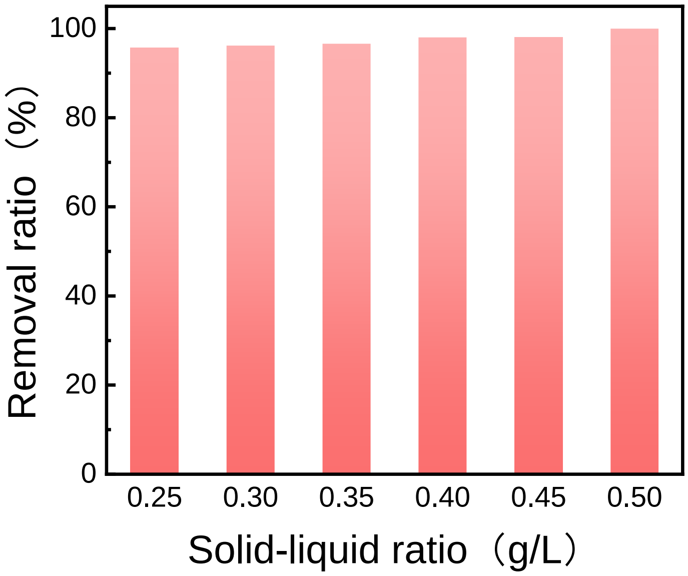

Figure S8. Effect of different solid-liquid ratio on uranium removal capacity of B-TiO_2@Co_2P-500.

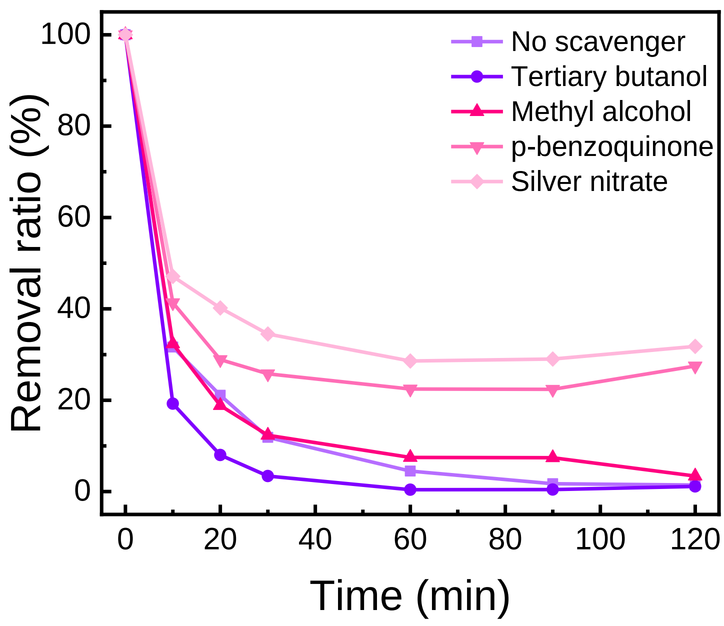

Figure S9. B-TiO_2@Co_2P-500's scavenger test results.

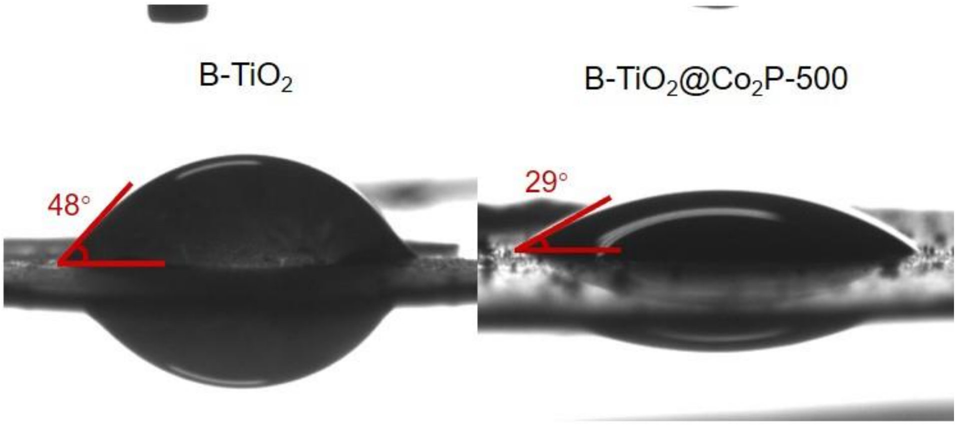

Figure S10. The static water contact angle of B-TiO_2 and B-TiO_2@Co_2P-500.

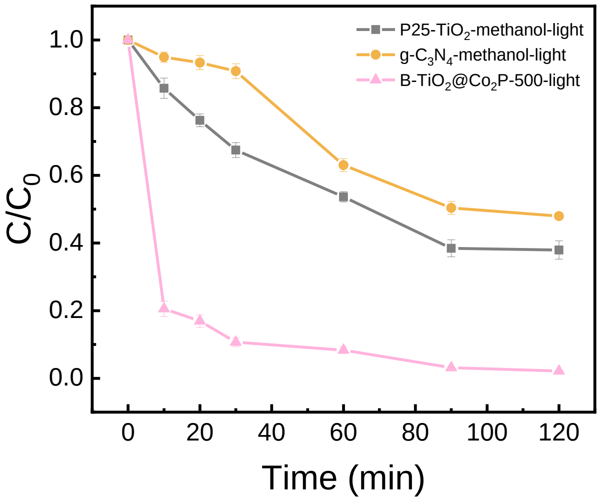

Figure S11. The test for commercial P25 and g-C_3N_4 and B-TiO_2@Co_2P-500 uranium removal performance under light conditions.

Table S1. The different catalysts for U(VI) photoreduction in recent literature.

Catalyst	Conditions	Electron sacrifice	Removal ratio	Ref.
B-TiO_2@Co_2P-500	C_0 = 50 ppm, m/V = 0.25 g/L, pH = 4, air atmosphere, 300 mW/cm^2	None	98% in 120 min	*This work*
AgNW/N-M(Ti)	C_0 = 100 ppm, m/V = 0.25 g/L, pH = 7, air atmosphere, 300 mW/cm^2	None	90.4% in 60 min	[S1]
Ag-C_3N_4/ $LaFeO_3$	C_0 = 0.1 mM, m/V = 0.2 g/L, pH = 5, air atmosphere, 300 mW/cm^2	7.5% Methanol	93.8% in 120 min	[S2]
SCU-19	C_0 = 400 ppm, m/V = 0.5 g/L, pH = 4,air atmosphere, 300 mW/cm^2	5% Methanol	91% in 2880 min	[S3]
PCN-222	C_0 = 400 ppm, m/V = 0.5 g/L, pH = 4, N_2 atmosphere, 350 mW/cm^2	10% Methanol	97% in 1440 min	[S4]
S-g-C_3N_4	C_0 = 30 ppm, m/V = 0.5 g/L, pH = 7, N_2 atmosphere, 350 mW/cm^2	2.5% Methanol	95% in 20 min	[S5]
CN550	C_0 = 200 ppm, m/V = 0.2 g/L, pH =	2 ml Methanol	97% in 360 min	[S6]

	5, air atmosphere, 200 mW/cm^2			
g-C_3N_4	C_0 = 20 ppm, m/V = 1.0 g/L, pH = 4, air atmosphere, 300 mW/cm^2	20 ppm Bisphenol A	90% in 30 min	[S7]
$WO_{2.78}$	C_0 = 8 ppm, m/V = 0.25 g/L, pH = 4.8, air atmosphere, 300 mW/cm^2	1 ppm Tannic acid	95% in 120 min	[S8]
BC-MoS_{2-x}	C_0= 8 ppm, m/V = 0.5 g/L, pH= 5, air atmosphere, 300 mW/cm^2	25 ppm Tannic acid	92% in 120 min	[S9]
g-C_3N_4/GO	C_0= 80 ppm, m/V = 0.1 g/L, pH= 5, air atmosphere, 300 mW/cm^2	25 ppm Tannic acid	91% in 40 min	[S10]
g-C_3N_4/TiO_2	C_0 = 20 ppm, m/V = 0.25 g/L, pH = 7, air atmosphere, 300 mW/cm^2	20 ppm As(III)	80% in 240 min	[S11]
C_3N_5/RGO	C_0 = 10 ppm, m/V = 0.2 g/L, pH = 5, air atmosphere, 300 mW/cm^2	None	94.9% in 100 min	[S12]
Fe_2O_3/GO	C_0 = 5 ppm, m/V = 1 g/L, pH = 4, air atmosphere, 300 mW/cm^2	None	76% in 180 min	[S13]
BCN	C_0 = 10 mM, m/V = 0.5 g/L, pH = 4, air atmosphere, 300 mW/cm^2	None	97.4% in 180 min	[S14]
TTT-DTDA	C_0 = 50 ppm, m/V = 1 g/L, pH = 5, air atmosphere, 300 mW/cm^2	None	98.2% in 720 min	[S15]
